# Cadmium-induced Oxidative Stress and Evaluation of *Embilica Officinalis* and Stressroak in Broilers

**DOI:** 10.4103/0971-6580.72669

**Published:** 2010

**Authors:** G. Swapna, A. Gopala Reddy, A. Rajasekhar Reddy

**Affiliations:** Department of Pharmacology and Toxicology, College of Veterinary Science, Rajendranagar, Hyderabad - 30, India; 1 Department of Pharmacology and Toxicology, College of Veterinary Science, Korutla, Karimnagar (Dist), Andhra Pradesh, India

**Keywords:** Cadmium, *Emblica officinalis*, oxidative stress, stressroak, vitamin E

## Abstract

Cadmium (Cd) toxicity was studied in broilers, and efficacy of *Emblica officinalis* (500 ppm in feed), vitamin E (300 ppm in feed), and stressroak (1 g/kg feed) were evaluated for prophylactic and therapeutic management of Cd toxicity. One-day-old male broiler chicks were randomly divided into eight groups consisting of 10 chicks in each. Groups 1 and 2 were maintained as plain control and Cd (100 ppm in feed) toxic control (for six weeks). Groups 3, 4, and 5 were maintained on a combination of Cd (100 ppm in feed) and *Emblica officinalis*, vitamin E, and stressroak for six weeks. Groups 6, 7, and 8 were maintained with Cd for the first four weeks and on *Emblica officinalis*, vitamin E, and stressroak during the subsequent two weeks without Cd. Body weights, feed consumed, Feed conversion ratio (FCR), and glulathione (GSH) were significantly (*P*<0.05) decreased, whereas the activities of antioxidant enzymes (catalase and Superoxide dismutase (SOD)) and concentration of Thiobarbituric acid reacting substances (TBARS) were significantly (*P*<0.05) increased in toxic control group. After treatment with *Emblica officinalis*, vitamin E, and stressroak in groups 6, 7, and 8 during last two weeks and discontinuation of Cd, the parameters revealed improvement. From this study, it is concluded that Cd induces toxicity by oxidative stress, and supplementing *Emblica officinalis*, vitamin E, and stressroak in feed is useful in preventing and treating the toxicity.

## INTRODUCTION

Cadmium (Cd) is a hazardous heavy metal that is widely distributed in the environment; it is present in trace levels in sea water and in a broad range of animal and plant species.[1] Many of the poultry industries are located near highways and in the proximity of industries that liberate Cd as pollutant. So, it is a common finding that poultry industry is affected with Cd toxicity. In poultry, diets containing 60 to 75 ppm Cd have been reported to induce reduced egg production and kidney damage.[2] Cd is a potent inducer of apoptosis; the apoptotic effects of Cd, at least in part, are mediated through induction of oxidative stress by inhibition of antioxidant enzymes particularly thiol-containing antioxidant enzymes.[3,4] Cd is known to cause stress by increasing lipid peroxidation or by changing intracellular glulathione (GSH) levels and to affect the unbiquinin/ATP dependent proteolytic pathway.[5] Keeping these toxic effects of Cd in view, an experimental study was conducted to evaluate its mechanisms of oxidative stress and to evaluate the benefit of using *Embilica officinalis*, vitamin E, and stressroak.

## MATERIALS AND METHODS

A total of 80 one-day-old male broiler chicks of Cobb strain obtained from Venkateshwara Hatcheries were randomly divided into eight groups consisting of 10 chicks in each. Group 1 was maintained on basal diet for six weeks, group 2 on Cd (100 ppm in feed) for six weeks, group 3 on a combination of Cd + *Emblica officinalis* (500 ppm in feed) for six weeks, group 4 on a combination of Cd + vitamin E (300 ppm in feed) for six weeks. Group 5 was fed on a combination of Cd + stressroak (Himachal Pradesh.) (1 g/kg feed) for six weeks. Groups 6, 7, and 8 were fed with Cd for first four weeks followed by *Emblica officinalis*, vitamin E, and stressroak, respectively during subsequent two weeks. Body weights of birds were recorded at weekly intervals to determine growth pattern and feed efficiency. The blood samples were drawn from wing vein at the end of fourth and sixth week for assay of SOD[6] and catalase.[7] TBARS[8] and GSH[9] were estimated in liver tissue at the end of sixth week. The data were analyzed by one-way ANOVA using statistical package for social sciences (SPSS) 10^th^ version. *P*<0.05 was considered as significant.

## RESULTS AND DISCUSSION

The average weekly body weight (g) of basal diet control (group 1) was 1795.2 ± 60.77 at the end of sixth week, and it was significantly (*P*<0.05) decreased to 1233.9 ± 40.28 in Cd toxic control group 2. The Cd toxic control groups 6, 7, and 8 that were treated with *Emblica officinalis*, vitamin E, and stressroak during the last two weeks, following discontinuation of Cd, revealed a significant (*P*<0.05) increase in the body weights (1355.5 ± 12.03, 1368.6 ± 30.84, and 1364.0 ± 42.92, respectively) as compared with the toxic control group 2 (1233.9 ± 40.28) at the end of sixth week. The progressive loss of body weight indicates growth-retarding effect of Cd, and this could be attributed to reduced feed intake[1] and general systemic toxaemia by Cd. Cd-induced oxidative stress plays a major role in decreasing the performance in broilers.[10] The results on performance parameters like body weights can be further substantiated from the altered enzymatic and nonenzymatic antioxidants in this study. *Emblica officinalis*, vitamin E, and stressroak supplementation in groups 6, 7, and 8 during the last two weeks improved the performance.

The weekly feed consumption (g) per bird in group 1 was 520 and 900, respectively at the end of fourth and sixth week, and the feed consumption of toxic control groups 2, 6, 7, and 8 at the end of fourth week was 396, 400, 408, and 412, respectively. Following supplementation of *Emblica officinalis*, vitamin E, and stressroak in groups 6, 7, and 8, respectively during the last two weeks after discontinuation of Cd revealed the feed intake as 886, 889, and 886, respectively, and these values were higher when compared with that of group 2. The FCR of group 1 was 1.40 and 2.19 respectively at the end of fourth and sixth week. The FCR of toxic control groups 2, 6, 7, and 8 was 1.72, 1.69, 1.74, and 1.77, respectively at the end of fourth week and it was higher as compared with that of group 1. However, following supplementation of *Emblica officinalis*, vitamin E, and stressroak respectively in groups 6, 7, and 8, there was a decrease in FCR, i.e., 2.27, 2.21, and 2.46, respectively at the end of sixth week as compared with group 2.

The activity of enzymatic antioxidant defenses in blood such as SOD (units/g protein) and catalase (moles/sec) was significantly (*P*<0.05) elevated in toxic controls (2, 6, 7, and 8) as compared with group 1 at the end of fourth week [Table 1]. On supplementation of *Emblica officinalis*, vitamin E, and stressroak in groups 6, 7, and 8 as a therapy, the antioxidant enzyme level in blood revealed a decreasing trend at the end of sixth week. The tissue (liver) GSH level was significantly (*P*<0.05) decreased in toxic control group 2 (75.857 ± 0.062) when compared with the remaining groups, whereas TBARS concentration in liver was significantly (*P*<0.05) increased in toxic control group 2 (1.253 ± 0.012) when compared with the remaining groups at the end of sixth week. Liver TBARS concentration and blood catalase and SOD activities were significantly increased in toxic group, suggesting the ongoing peroxidative stress. It has been reported earlier that Cd impairs the antioxidant defenses of cells and make them more susceptible to oxidative attacks.[11] Cd induces a time- and dose-dependent decrease in intracellular glutathione.[5]

**Table 1:** Results of cadmium-induced oxidative stress

Group	Catalase (moles/s)		SOD (units/g protein)		TBARS (n moles/mg protein)	GSH (n moles/g protein)
	4^th^ week	6^th^ week	4^th^ week	6^th^ week	6^th^ week	6^th^ week
Basal diet (1 – 42 d)	2.365 ± 0.050^aA^	2.515 ± 0.085^aB^	41.555 ± 0.488^aA^	43.422 ± 0.049^aB^	0.805 ± 0.047^a^	105.25 ± 0.241^e^
Cadmium (1 – 42 d)	3.387 ± 0.075^cA^	3.626 ± 0.064^eB^	60.845 ± 0.021^efA^	70.115 ± 0.014^eB^	1.253 ± 0.012^e^	75.857 ± 0.062^a^
Cadmium + *Emblica officinalis* (1 – 42 d)	2.465 ± 0.030^bA^	2.653 ± 0.047^bB^	55.090 ± 0.010^cA^	57.135 ± 0.043^dB^	0.954 ± 0.085^d^	90.575 ± 0.064^c^
Cadmium + vitamin E (1 – 42 d)	2.453 ± 0.086^bA^	2.668 ± 0.048^cB^	55.072 ± 0.047^cA^	57.087 ± 0.085^cdB^	0.953 ± 0.086^d^	90.617 ± 0.025^c^
Cadmium + stressroak (1 – 42 d)	2.464 ± 0.085^bA^	2.665 ± 0.043^cB^	54.077 ± 0.050^bA^	56.135 ± 0.047^bB^	0.955 ± 0.057^d^	95.677 ± 0.047^d^
Cadmium (1 – 28 d); *Emblica officinalis* (29 – 42 d)	3.385 ± 0.025^cA^	2.856 ± 0.031^dB^	61.077 ± 0.012^fA^	57.062 ± 0.015^cB^	0.865 ± 0.011^c^	85.667 ± 0.094^b^
Cadmium (1 – 28 d); vitamin E (29 – 42 d)	3.386 ± 0.040^cA^	2.855 ± 0.057^dB^	60.080 ± 0.025^dA^	57.077 ± 0.025^cB^	0.864 ± 0.025^c^	85.915 ± 0.025^b^
Cadmium(1 – 28 d); stressroak (29 – 42 d)	3.385 ± 0.028^cA^	2.854 ± 0.054^dB^	60.350 ± 0.243^cdA^	57.085 ± 0.085^dcB^	0.856 ± 0.085^b^	85.672 ± 0.021^b^

Values are mean + SE of six observations; Means with different alphabets as superscripts differ significantly (*P*<0.05)-ANOVA; Capital alphabets (horizontal comparison); small alphabets (vertical comparison); SOD - ; TBARS - ; GSH – glulathione; SE - Standard error

Stressroak is a polyherbal formulation comprising of *Withania somnifera, Ocimum sanctum, Mangifera indica*, and shilajit. Lupeol, a triterpene present in mango and other fruits, was found to be effective in combating oxidative stress-induced cellular injury of mouse liver by modulating cell-growth regulators.[12] The aqueous stem bark extract of *Mangifera indica L*. has been reported to have antioxidant properties.[13] Vitamin E which is abundant in several natural sources has free radical-quenching activity,[14] by decreasing MDA concentration and increasing the activity of liver catalase and brain SOD.[15] The activity of the fruit of *Emblica officinalis* was attributed to the high-content of ascorbic acid (1.28%, w/w), and vitamin C accounts for approximately 45 to 70% of the antioxidant activity.[16] The tannoid principles of fruits of amla, comprising emblicanin A, emblicanin B, punigluconic, and pedunculagin are reported to exhibit antioxidant activity *in vitro* and *in vivo*.[17] Kumar *et al*.[18] studied the free and bound phenolic antioxidants in amla and attributed higher level of antioxidant activity in *Emblica officinalis* to the phenolic content. Gallic acid and geraniin, which are the active principles of *Phyllanthus emblica*, have been reported to possess strong nitric oxide (free radical) scavenging activity.[19]

## References

[1] Uyanik F, Even M, Atasever A, Tuncoku G, Kolsuz AH (2001). Changes in some Biochemical Parameters and organs of broilers exposed to cadmium and effect of zinc on cadmium induced alterations. Israel J Vet Med.

[2] Ammerman Trace Mineral and Cadmium Toxicity. http://wwwsaltinstititeorg.

[3] Liochev SI, Sigel A, Sigel H (1999). The mechanism of “Fenton-like reactions and their importance for biological systems. A biologists’ view. Metal Ions in Biological Systems.

[4] Watjen W, Beyersmann D (2004). Cadmium induced apoptosis in C6 glioma cells: Influence of oxidative stress. Biometals.

[5] Figueiredo-Pereira ME, Yakushin S, Cohen G (1998). Disruption of the intracellular sulfhydryls homeostasis by cadmium induced oxidative stress leads to Protein thiolation and ubiquitination in Neuronal cells. J Biol Chem.

[6] Marklund S, Marklund G (1974). Involvement of the superoxide anion radical in the autooxidation of pyrogallol and a convenient assay for superoxide dismutase. Environ J Biochem.

[7] Britton C, Machlly AC (1956). Assay of catalase and peroxidases. Methods Enzymol.

[8] Subramanian KA, Manohar M, Mathan VI (1988). An unidentified inhibitor of lipid peroxidation in intestinal mucosa. Biochim Biophysic Acta.

[9] Moron MS, Depierre JW, Mannervik B (1979). Levels of glutathione, glutathione reductase and glutathione - s- transferase in rat lung and liver. Biochim Biophysic Acta.

[10] Erdogan Z, Erdogan S, Celk S, Unlu A (2005). Effects of ascorbic acid on cadmium induced oxidative stress and performance of broilers. Biol Trace Ele Res.

[11] Ikediobi CO, Badisa VL, Ayuk-Takem LT, Latinwo LM, West J (2004). Response of antioxidant enzymes and redox metabolites to cadmium induced oxidative stress in CRL-149 normal rat liver cells. Intl J Mol Med.

[12] Prasad S, Kalra N, Shukla Y (2007). Hepatoprotective effects of lupeol and mango pulp extract of carcinogen induced alteration in Swiss albino mice. Mol Nutr Food Res.

[13] Hernandez P, Delgado R, Walczak H (2006). *Mangifera indica* L. extract protects T cells from activation-induced cell death. Int Immunopharmacol.

[14] Halim EM, Mukhopadhyay AK (2006). Effect of *Ocimum sanctum* (Tulsi) and vitamin E on biochemical parameters and retinopathy in streptozotocin induced diabetic rats. Indian J Clin Biochem.

[15] Lin YF, Tsai HL, Lee Y, Chang SJ (2005). Maternal Vit E supplementation affects the antioxidant capability and oxidative status of hatching chicks. J Nutrition.

[16] Scartezzini P, Antogoni F, Raggi MA, Poli F, Sabbioni C (2006). Vitamin C content and antioxidant activity of the fruit and of the Ayurvedic preparation of *Emblica officinalis* Gaertn. J Ethnopharmacol.

[17] Bhattacharya A, Ghosal S, Bhattacharya SK (2000). Antioxidant activity of tannoid principles of *Emblica officinalis* (amla) in chronic stress induced changes in rat brain. Indian J Exp Biol.

[18] Kumar GS, Harish N, Dharmesh SM, Salimath PV (2006). Free and bound phenolic antioxidants in amla (*Emblica officinalis*) and turmeric (*Curcuma longa*). J Food Compost Anal.

[19] Kumaran A, Karunakaran RJ (2006). Nitric oxide radical scavenging active component from *Phyllanthus emblica* L. Plant Foods Hum Nutr.

